# Intraoperative cardiac arrest caused by unexpected vasospastic angina requiring prolonged resuscitation using extracorporeal membrane oxygenation: a case report

**DOI:** 10.1186/s40981-023-00667-z

**Published:** 2023-11-09

**Authors:** Shinji Sugita, Masanobu Obata, Fumihiko Hasunuma, Atsuhiro Sakamoto

**Affiliations:** 1 Department of Anesthesiology, Nippon Medical School Musashi-kosugi Hospital, 1-383 Kosugi-cho, Nakahara-ku, Kawasaki-shi, Kanagawa, 211-8533 Japan; 2 Department of Anesthesiology and Pain Medicine, Graduate School of Medicine, Nippon Medical School, 1-1-5 Sendagi, Bunkyo-ku, Tokyo, 113-8602 Japan

**Keywords:** Intraoperative cardiac arrest, Vasospastic angina, Extracorporeal membrane oxygenation, Monitoring

## Abstract

**Background:**

Vasospastic angina (VSA) occurring during surgery is rare but can lead to sudden intraoperative cardiac arrest.

**Case presentation:**

A 77-year-old man with hypertension, and no history of coronary artery disease, displayed an abrupt ST-segment elevation on the electrocardiogram (ECG) during laparoscopic inguinal hernia surgery under general anesthesia. Subsequently, ventricular fibrillation (VF) occurred, with a finding suggesting ischemic myocardial contracture by transesophageal echocardiography. VF was refractory to cardiopulmonary resuscitation (CPR), and veno-arterial extracorporeal membrane oxygenation (VA ECMO) was introduced. Spontaneous circulation resumed 77 min post-cardiac arrest. VSA was confirmed through the patient’s clinical course and coronary angiography. Subsequently, VA ECMO was terminated, and the patient was discharged uneventfully.

**Conclusions:**

Extracorporeal CPR may be a valuable alternative to extended resuscitation for refractory ventricular arrhythmias by VSA.

## Background

Vasospastic angina (VSA) arises when a coronary artery vasospasm leads to a decreased blood supply to the myocardium [1]. It is typified by heart attacks that transpire at rest, especially during nighttime and early mornings. However, it can also manifest intraoperatively. A prior study indicated that 18 out of 42 patients, who underwent coronary catheterization due to intraoperative cardiovascular events among a total of 77,745 patients undergoing non-cardiac surgeries, were diagnosed with VSA [2]. In certain scenarios, VSA might culminate in cardiac arrest, making its management during resuscitation challenging [3].

Extracorporeal CPR (ECPR), a resuscitation approach employing extracorporeal membrane oxygenation, is contemplated when standard resuscitation proves ineffective [4–8]. Even though intraoperative cardiac arrest occurrences are exceedingly rare, ECPR can be a therapeutic recourse.

In this paper, we detail a case of an intraoperative cardiac arrest instigated by an unforeseen VSA that necessitated extended resuscitation via ECPR. We also illustrate the variation in the depth of anesthesia values during resuscitation and deliberate on both the quality of resuscitation and the prognostic implications.

## Case presentation

**Fig. 1:**
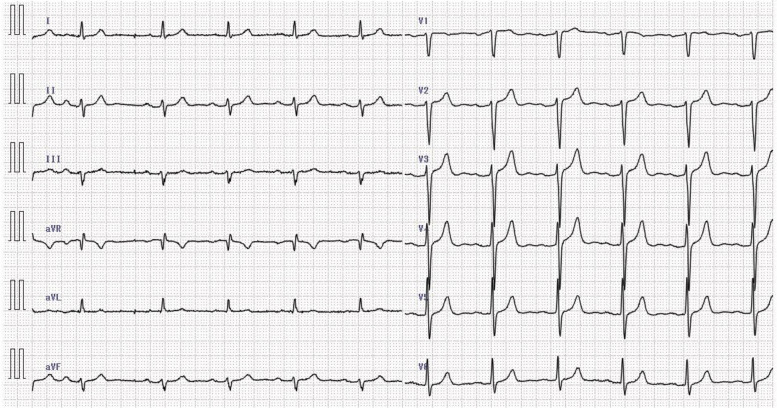
Preoperative electrocardiogram

A 77-year-old male, measuring 170 cm and weighing 64.7 kg, was slated for a laparoscopic inguinal hernia operation under general anesthesia, complemented with a peripheral nerve block. His medical history included hypertension, and he was on amlodipine and candesartan. He consumed alcohol minimally and had a smoking history of 10 cigarettes per day from the age of 20 to 30. Preoperative 12-lead electrocardiogram (ECG) showed a first-degree atrioventricular node block and left axis deviation (Fig. 1). The preoperative revised cardiac risk index was scored at 0 out of a possible 6 points [9].

Anesthesia was induced with 100 mg propofol, 150 μg fentanyl, 0.3 μg/kg/min remifentanil, and 60 mg rocuronium and maintained with 1 L/min oxygen, 2 L/min air, 1.5% sevoflurane, and 0.1−0.2 μg/kg/min remifentanil. Additionally, 100 μg bolus fentanyl and 10 mg rocuronium were administered as needed. The SEDLINE® system (Masimo Corporation; Irvine, CA, USA) was used to monitor the depth of anesthesia, ensuring the patient state index (PSi) remained between 25 and 50. A standard 3-lead ECG was continually monitored. Prior to the surgery, an ultrasound-guided rectus sheath block was administered with 50 mL of 0.125% levobupivacaine. Ephedrine, in 4 or 8 mg boluses, was administered as needed for intraoperative hypotension.

**Fig. 2:**
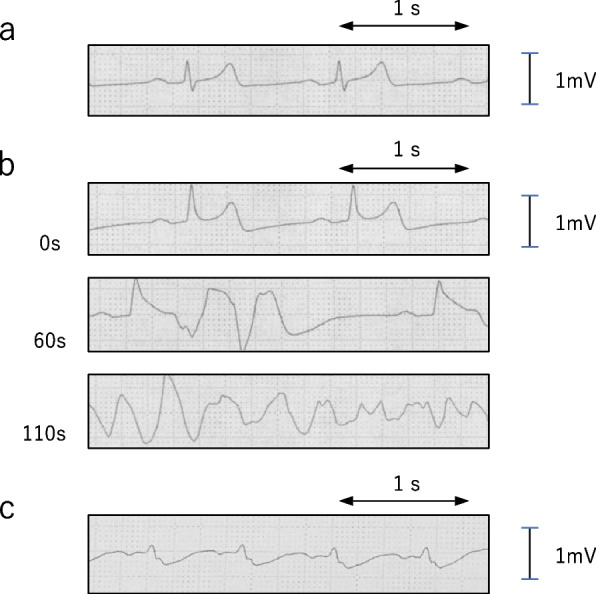
Electrocardiogram (ECG) during surgery. Initial ECG shows no abnormalities **a** The ST segment elevated, followed by the emergence of ventricular fibrillation after 110 s **b** Spontaneous circulation returned 77 min post-cardiac arrest **c**

The surgery proceeded without complications. Immediately before completing mesh fixation, a sudden ST elevation appeared on the ECG, with a heart rate of 46 bpm, blood pressure of 102/50 mmHg, peripheral oxygen saturation of 98%, end-tidal carbon dioxide tension (EtCO_2_) of 40 mmHg, and PSi of 35. After 110 s, ventricular fibrillation (VF) manifested on the ECG, leading to cardiac arrest (Fig. 2). The attending anesthesiologist swiftly notified the available physicians. The laparoscopic procedure was halted, all anesthetic agents were discontinued, and cardiopulmonary resuscitation (CPR) was initiated immediately based on the Advanced Cardiovascular Life Support algorithm. Despite four attempts at defibrillation, the VF persisted. Transesophageal echocardiography detected no significant anomalies except for ischemic myocardial contracture. Given the patient’s non-response to 150 mg of amiodarone and ongoing CPR, extracorporeal CPR (ECPR) was initiated for this refractory lethal arrhythmia.

**Fig. 3:**
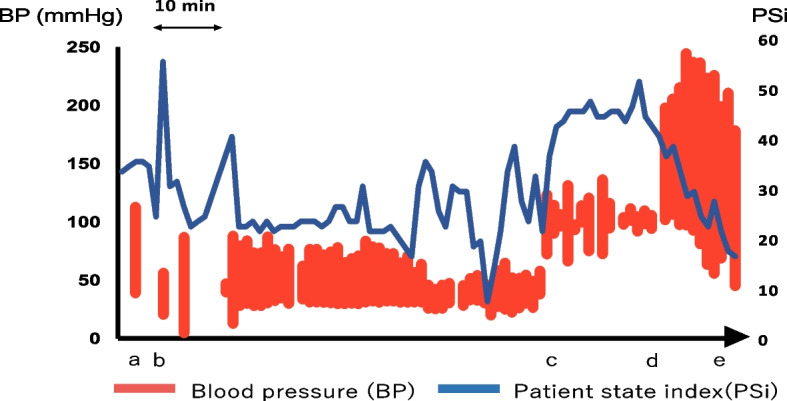
Parameter fluctuations during resuscitation. Following an abrupt ST-T wave elevation, the electrocardiogram indicated ventricular fibrillation leading to cardiac arrest **a** The laparoscopic procedure and general anesthesia were halted, and standard cardiopulmonary resuscitation was initiated **b** The patient state index (PSi) value was elevated after the commencement of veno-arterial extracorporeal membrane oxygenation **c** Spontaneous circulation was restored 77 min post-cardiac arrest upon the fifth defibrillation attempt **d** Resumption of anesthetic agents resulted in a decline in the PSi value **e** ABP, arterial blood pressure

Veno-arterial extracorporeal membrane oxygenation (VA ECMO) was initiated 58 min post-cardiac arrest. Return of spontaneous circulation was achieved 77 min post-arrest after the fifth defibrillation attempt. In total, 18 mg of adrenaline was administered. During CPR, along with ECG, non-invasive blood pressure, arterial blood pressure, EtCO_2_, and PSi were consistently monitored. Arterial blood samples were collected for gas analysis. The EtCO_2_ was approximately 2–24 mmHg, while the PSi values ranged from 15 to 38, spiking to 50 post-VA ECMO initiation (Fig. 3).

**Fig. 4:**
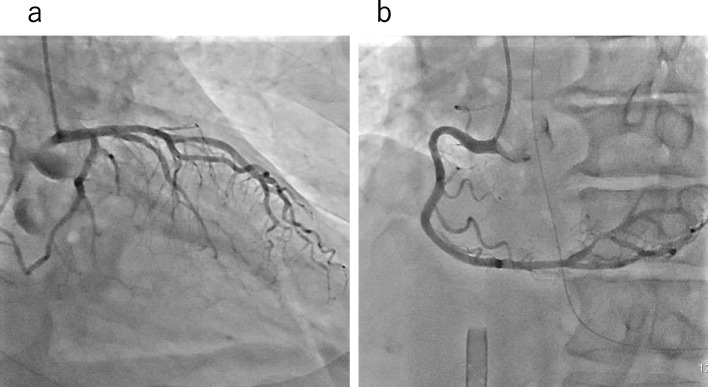
Post-resuscitation coronary arteriography. Both the left **a** and right **b** coronary arteries showed no significant stenosis

Coronary angiography after resuscitation in the angiography room displayed no significant coronary artery stenosis (Fig. 4), leading to a diagnosis of VSA, along with the clinical course. The patient was returned to the operating room, and the surgery was completed under ECMO management. No arrhythmia was observed under continuous administration of amiodarone 50 mg/h and diltiazem 1 μg/kg/min.

After the operation, the patient was admitted to the intensive care unit. A thorough trans-thoracic echocardiography was performed but no abnormal finding including ischemic regional wall motion abnormality was observed. Targeted temperature management (TTM) was performed to set at 35–36 °C [10, 11]. A brain computed tomography scan revealed no discernible damage from the prolonged resuscitation. ECMO support was ceased a day post-surgery, with TTM continued for 72 h. Consciousness was reestablished, and the patient was relocated to the general ward 8 days after surgery. Oral diltiazem 100 mg/day was initiated as treatment for VSA.

No subsequent residual central nervous system (CNS) symptoms, new arrhythmias, or ECG changes were noted. The consideration was given to implant an implantable cardioverter-defibrillator based on treatment guidelines [12]. Nonetheless, the patient was discharged 28 days post-surgery, choosing against the procedure, given that the incident occurred under general anesthesia. At the 1-year follow-up, he displayed no arrhythmia-related events or apparent CNS symptoms.

## Discussion

We present a case of intraoperative cardiac arrest attributable to VSA, which was successfully resuscitated using ECMO. VSA is associated with several risk factors, such as smoking, alcohol consumption, abnormal lipid metabolism, stress, and inflammation. However, predicting VSA remains challenging [1, 13]. Numerous cardiac risk assessments for noncardiac surgery have been proposed. For example, the revised cardiac risk index, a frequently utilized scoring system with 6 criteria, indicates that scores of 0 or 1 are associated with a perioperative cardiovascular event rate of 0.4% or 0.9%, respectively, while a score of 3 points corresponds to an event rate of 6.6% [9]. Notably, VSA can occur even in patients with low scores [2]. From the perspective of intraoperative risk factors, anesthetic agents and hypertensive medications, such as phenylephrine and ephedrine, have been implicated in VSA onset [14, 15]. Moreover, Koshiba et al. observed that factors like inadequate anesthesia depth, vasopressor use, vagal reflexes, and epidural blocks were potential triggers [16]. In the case discussed, VSA signs did not immediately follow hypotensive episodes or ephedrine administration, and no other discernible triggers were identified. In terms of treatment, agents like nitroglycerin and calcium channel blockers might have been beneficial. Furthermore, nitroglycerine can serve as a diagnostic therapeutic tool [17]. We were unable to prevent the cardiac arrest because of the swift progression of symptoms. This underscores the importance of suspecting VSA during intraoperative sudden ST elevations occurs in patients without discernible triggers or histories to ensure timely intervention.

Literature offers varying prognosis accounts concerning intraoperative cardiac arrests [18, 19]. While prompt identification and CPR are crucial, performing CPR on anesthetized patients presents challenges. As per guidelines on managing intraoperative cardiac arrests, ECPR becomes a consideration in refractory cases [20–22]. Given potential complications with ECMO, such as bleeding, its indication mandates careful consideration [8, 23]. Yet, when risks are minimized, ECMO can be therapeutically invaluable.

Studies suggest that ECPR is expected to improve low-flow time [24, 25]. Notably, in our case, PSi was elevated after the initiation of ECMO, hinting at better cerebral perfusion. PSi, calculated from a simultaneous four-channel electroencephalography derivation, is typically used during general anesthesia [26, 27]. While correlations between anesthesia depth and postoperative neurologic outcomes have been previously reported, the role of neurological monitoring during resuscitation in clinical settings remains controversial [28]. As this is a single case report, the findings might have limitations. Larger observational studies might be needed to validate the efficacy of monitoring anesthesia depth during resuscitation.

In conclusion, intraoperative VSA can lead to refractory lethal arrhythmias. While anticipating such incidents in patients with no prior history remains challenging, clinicians should be vigilant about VSA as a potential cause of intraoperative cardiac arrest. In select cases, ECPR might offer an advantageous approach over extended resuscitation.

## Data Availability

The datasets used and/or analyzed during the current study are available from the corresponding author upon reasonable request.

## Abbreviations

VSA: Vasospastic angina
ECG: Electrocardiogram
VF: Ventricular fibrillation
CPR: Cardiopulmonary resuscitation
ECPR: Extracorporeal cardiopulmonary resuscitation
VA ECMO: Veno-arterial extracorporeal membrane oxygenation
PSi: Patient state index
EtCO_2_: End-tidal CO_2_
TTM: Targeted temperature management
CNS: Central nervous system
